# Micro-Gas Flow Sensor Utilizing Surface Network Density Regulation for Humidity-Modulated Ion Transport

**DOI:** 10.3390/gels11080570

**Published:** 2025-07-23

**Authors:** Chuanjie Liu, Zhihong Liu

**Affiliations:** 1 School of Chemistry and Chemical Engineering, Beijing Institute of Technology, Beijing 100081, China; 7520230176@bit.edu.cn; 2 School of Digital Media, Shenzhen Polytechnic University, Shenzhen 518055, China

**Keywords:** surface effect, resistance response, gas flow sensor

## Abstract

As a bridge for human–machine interaction, the performance improvement of sensors relies on the in-depth understanding of ion transport mechanisms. This study focuses on the surface effect of resistive gel sensors and designs a polyacrylic acid/ferric ion hydrogel (PAA/Fe^3+^) gas flow sensor. Prepared by one-pot polymerization, PAA/Fe^3+^ forms a three-dimensional network through the entanglement of crosslinked and uncrosslinked PAA chains, where the coordination between Fe^3+^ and carboxyl groups endows the material with excellent mechanical properties (tensile strength of 80 kPa and elongation at break of 1100%). Experiments show that when a gas flow acts on the hydrogel surface, changes in surface humidity alter the density of the network structure, thereby regulating ion migration rates: the network loosens to promote ion transport during water absorption, while it tightens to hinder transport during water loss. This mechanism enables the sensor to exhibit significant resistance responses (ΔR/R_0_ up to 0.55) to gentle breezes (0–13 m/s), with a response time of approximately 166 ms and a sensitivity 40 times higher than that of bulk deformation. The surface ion transport model proposed in this study provides a new strategy for ultrasensitive gas flow sensing, showing potential application values in intelligent robotics, electronic skin, and other fields.

## 1. Introduction

As a bridge for human–machine interaction, sensors are profoundly reshaping the communication model between humans and machines. Like sensitive sensory tentacles, they can accurately capture various pieces of information in the physical world, such as light intensity, temperature changes, pressure magnitude, and human movements, and rapidly convert these analog signals into digital signals for transmission to machine systems [1,2,3,4]. Novel tactile sensors further enrich the dimensions of human–machine interaction: when users interact with touchscreen devices, they can precisely sense information such as touch force and texture, making virtual operations more tactile [5,6,7]. Common mechanical sensors mainly include strain sensors, capacitive sensors, inductive sensors, etc. Strain sensors exhibit enormous application potential in multiple fields such as memory devices, neuromorphic computing, and biosensing due to their simple structure, relatively easy fabrication process, and low cost [8,9]. At present, the sensitivity of resistive sensors is primarily improved through multiple approaches: adding nanocomposites such as carbon nanotubes and graphene [10,11,12]; designing and preparing materials with ordered microstructures, such as directionally aligned nanofibers and nanowire arrays [13,14,15]; and using 3D printing to design special structures like auxetic and porous structures [16,17,18].

In numerous studies on resistive sensors, the selection of low-modulus gel materials is currently the most widely studied. Low-modulus gel materials typically have a loose internal structure with weak constraints between molecular chains, containing more free spaces and pores. This structure provides relatively broad channels for ion migration, facilitating ion diffusion [19]. Therefore, many studies focus on selecting different gelling agents and fillers or modifying preparation methods to enable gels to form more and larger pores with improved connectivity. Alternatively, at the cost of mechanical strength and stability, reducing the number of crosslinking agents or choosing those with low crosslinking activity loosens the gel’s network structure, thereby decreasing the hindrance to anion–cation transport [7,20,21,22,23]. Although these works have contributed to the study of ion transport mechanisms in strain sensors, most focus on enhancing ion migration within the gel bulk under deformation, ignoring the surface effect of gels. The surface accounts for a small proportion of the total mass and is often overlooked, but when the hydrogel thickness decreases to the micrometer scale (<10 μm), the surface proportion can exceed 30%. The ordered molecular arrangement (such as the lamellar structure induced by the gas–liquid interface) reduces the activation energy for ion migration [24,25]. The thinning of hydrogel films significantly increases the specific surface area of sensing materials, making water molecule adsorption/desorption more efficient and highlighting surface effects [26,27].

Additionally, during gel synthesis, obvious differences between the surface and interior confirm that the formation of the gel’s gas–liquid surface is directional and templated. This surface templating effect creates a highly ordered structure, unlike the random topological structure inside [28]. Evidently, as polymers with unique physicochemical properties, gels have surfaces that are not merely simple boundaries—their molecular arrangement, charge distribution, and interaction patterns with ambient molecules significantly influence overall performance. It is particularly important and meaningful to achieve resistive changes in response to mechanical actions (micro-deformations or gentle breezes) without obvious deformation, similar to human skin. This imposes strict requirements on gas flow sensors in terms of sensitivity, response time, detection threshold, and range [29,30,31]. Airflow sensors have made remarkable research progress in recent years. The application of nanomaterials has become increasingly widespread, and their unique physical and chemical properties have effectively enhanced the sensitivity and response speed of sensors. For example, the team led by Zhang et al. [32] from Tsinghua University gave full play to the advantages of ultra-long carbon nanotubes and proposed the idea of preparing ultrasensitive airflow sensors with suspended networks. The trend of miniaturization and integration has become increasingly prominent, facilitating applications in various complex scenarios, especially in portable devices and micro-electromechanical systems. The new airflow sensor developed by the team led by Li et al. [33] from South China Agricultural University adopts a unique polygonal prism structure design, which is more sensitive to changes in wind speed, and uses artificial neural network technology to calculate the speed and direction of airflow. The ultra-high-resolution flexible flow field sensing system researched by the team led by Jiang et al. [34] from Beihang University is based on the calorimetric principle. A constant temperature difference is generated by a central micro-heater, and the surrounding thermistor array measures the temperature distribution. The temperature difference measured by the thermistor array can accurately reflect the flow velocity and direction. However, traditional airflow sensors based on hot-wire or hot-film mechanisms have defects such as complex manufacturing processes (highly dependent on MEMS chips), high power consumption, rough structural design, and vulnerability to damage [35,36,37,38]. Among airflow sensing systems with multiple working mechanisms, resistive sensors have become a highly promising candidate due to their simple manufacturing process and convenient signal acquisition [39,40].

**Figure 1:**
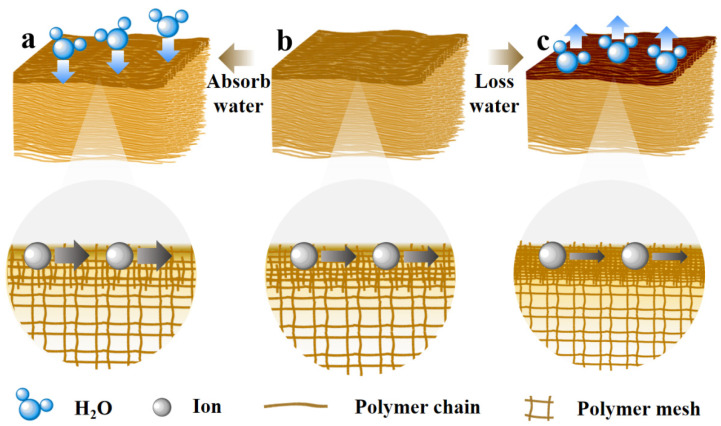
A schematic diagram of the sensing mechanism of the hydrogel. (**a**) The ion migration process on the surface of the water-absorbing hydrogel; (**b**) the ion migration process on the surface of the normal hydrogel; (**c**) the ion migration process on the surface of the water-losing hydrogel.

This study provides a detailed introduction to the surface ion transport phenomenon in resistive gel sensors, aiming to deeply understand the charge transport behavior of ions in hydrogel networks and propose a surface response mechanism based on ion transport regulation. Based on the decisive role of surface molecular arrangement in hydrogels for charge transport, an ultrasensitive regulation strategy for gel surfaces modulated by water molecule migration is developed, where a class of highly sensitive gas flow sensors is designed. The sensing mechanism is shown in Figure 1: when a gas flow passes over the hydrogel surface, a series of chain reactions are triggered. The gas flow alters the humidity conditions at the hydrogel surface, which in turn causes changes in the density of the surface network, significantly affecting ion migration rates and transport pathways. When the hydrogel surface absorbs water, the surface network structure loosens, facilitating ion migration (Figure 1a); when the surface loses water, the network structure tightens, hindering ion migration (Figure 1c). As charge carriers, changes in ion transport can be rapidly converted into detectable electrical signals. When ion migration rates or pathways are altered by gas flow, the charge distribution and conductivity of the hydrogel surface change accordingly.

**Figure 2:**
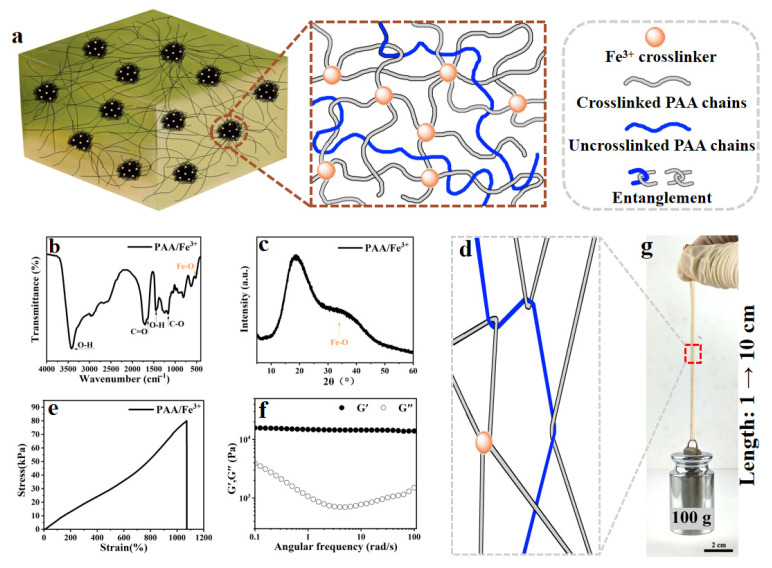
The preparation and properties of the PAA/Fe^3+^ hydrogel. (**a**) Schematic diagrams of the PAA/Fe^3+^ hydrogel based on crosslinked and uncrosslinked PAA chains. (**b**) FT-IR spectra (400–4000 cm^−1^) of the PAA/Fe^3+^ hydrogel. (**c**) The XRD curve (5–60°) of the PAA/Fe^3+^ hydrogel. (**d**) The stretchable topological network. (**e**) The stress–strain curves of the PAA/Fe^3+^ hydrogel. (**f**) The storage modulus (G′) and loss modulus (G″) of the PAA/Fe^3+^ hydrogel. (**g**) A photograph of the loading process (loading weight: 100 g) of a cuboid PAA/Fe^3+^ hydrogel.

Therefore, this study designs and develops a polyacrylic acid-based hydrogel (PAA/Fe^3+^) using a one-pot method, where crosslinked and uncrosslinked PAA chains collectively construct the polymer network (as shown in Figure 2). By placing electrodes on the hydrogel surface, changes in electrical signals can be rapidly captured. The PAA/Fe^3+^ surface can recognize resistance responses from non-mechanical deformations: a gentle breeze produces a ΔR/R_0_ of 0.03, while continuous blowing yields a ΔR/R_0_ of 0.5—far higher than the ΔR/R_0_ of 0.0008 caused by mechanical displacement deformation. Focusing on resistance changes induced by surface molecular conformational changes, the response feedback can be achieved with minimal energy input. It is hoped that this work will provide new insights into the surface ion migration mechanism of hydrogels and contribute to the development of gas flow sensors.

## 2. Results and Discussion

### 2.1. PAA/Fe^3+^ Hydrogel Design and Preparation

The hydrophilic groups in the molecular structure of acrylic acid (CH_2_=CHCOOH) endow polyacrylic acid hydrogels with good hydrophilicity. A large number of carboxyl functional groups on the main chain are partially ionized in aqueous solutions to generate directionally mobile ions. Their low glass transition temperature imparts strong mobility to polymer segments, promoting ion diffusion and transport in the polymer network, thereby enhancing ion mobility, endowing good electrical conductivity, and enabling the rapid and accurate sensing of changes and signal conversion. Excellent water retention properties maintain the stability of the network structure, and self-healing properties improve durability. In addition, as a common chemical raw material, acrylic acid has a low cost, making hydrogels prepared from acrylic acid have broad potential application prospects in many fields such as medicine, agriculture, and industry, and conducive to large-scale production [41,42].

The preparation mechanism of the PAA/Fe^3+^ hydrogel proposed in this paper is shown in Figure 2a. A hydrogel with uniform texture and good performance was prepared by one-pot polymerization. AA monomers undergo free radical polymerization under the action of initiator potassium persulfate (KPS) to form long PAA chains. Carboxyl groups on part of the PAA chains complex and crosslink with Fe^3+^, and the crosslinked and uncrosslinked PAA chains entangle with each other to form a tight three-dimensional (3D) network structure, ensuring the hydrogel can be freely customized in shape (Figure S1).

Fourier transform infrared spectroscopy (FT-IR) and X-ray diffraction (XRD) were performed on the PAA/Fe^3+^ hydrogel to characterize the complex crosslinking between carboxyl groups and Fe^3+^ within the hydrogel, as shown in Figure 2b,c. The PAA/Fe^3+^ hydrogel exhibits characteristic absorption bands near 3422, 1649, 1166, and 1452 cm^−1^, corresponding to O–H stretching, C=O stretching, C–O stretching, and O–H bending vibrations [43,44]. An absorption peak of stretching vibration from Fe–O coordination bonds appears at around 500–600 cm^−1^, directly confirming the coordination interaction between Fe^3+^ and carboxyl groups of PAA. The position and number of peaks in the XRD pattern (Figure 2c) indicate that the PAA/Fe^3+^ hydrogel has an amorphous structure, with broad peaks suggesting relatively disordered molecular chain arrangements. The XRD pattern shows a coordination structure of Fe–O–PAA, and a diffraction peak corresponding to Fe–O coordination compounds appears at 30–50°, which is attributed to the formation of new complex structures between Fe^3+^ and carboxyl groups of PAA.

In this study, a structurally rational topological molecular network was constructed using acrylic acid as the raw material (Figure 2a). Polyacrylic acid (PAA) molecular chains are interconnected and entangled through abundant intermolecular hydrogen bonds, electrostatic interactions, and metal coordination bonds. These conformations act as sacrificial configurations for energy dissipation during hydrogel deformation (Figure 2d). As shown in Figure 2e, the prepared hydrogel exhibits excellent tensile properties, with an elongation exceeding ten times its original length and displaying distinct linear regions in the tensile curve. These linear regions reflect differences in mechanical behavior at the molecular chain level: the initial linear segment is associated with the elastic stretching and flexible adjustment of uncrosslinked chains, while with increasing tensile strain, the transition to another linear segment corresponds to the synergistic stress-bearing of the crosslinked network, where sacrificial bonds (such as hydrogen bonds and coordination bonds) gradually break to dissipate energy, while the molecular backbones maintain rigid support, demonstrating the characteristic of combining rigidity and flexibility.

By comparing tensile and rheological data, it is found that the PAA/Fe^3+^ hydrogel exhibits a high storage modulus (Figure 2f) and appropriate loss factor (Figure S2) in rheological tests, and its tensile Young’s modulus conforms to Hooke’s law (Figure 2e), confirming the coexistence of rigidity and flexibility in the molecular chains. An effective energy-dissipating crosslinked network is formed between the molecular chains, and this relatively regular crosslinked structure enables the material to maintain good elasticity under dynamic loading. Meanwhile, dense PAA chain entanglements around crosslinking points contribute to mechanical strength: the hydrogel achieves a tensile strength of 80 kPa and a breaking elongation of 1100%, capable of easily lifting a 100 g weight (Figure 2g). Thus, the topological molecular network constructed in this study features interconnected and entangled crosslinked and uncrosslinked PAA chains via abundant intermolecular hydrogen bonds and electrostatic interactions (Figure 2a,d). These conformations act as sacrificial configurations for energy dissipation during hydrogel deformation (Figure 2d), and the mutual validation of tensile and rheological data confirms the synergistic effect of rigidity and flexibility in the topological network—ensuring mechanical performance through dense chain entanglements while maintaining elasticity under dynamic loads via regular crosslinked structures, embodying the consistency of structure and performance.

### 2.2. Airflow Sensitiviy of PAA/Fe^3+^ Hydrogel

**Figure 3:**
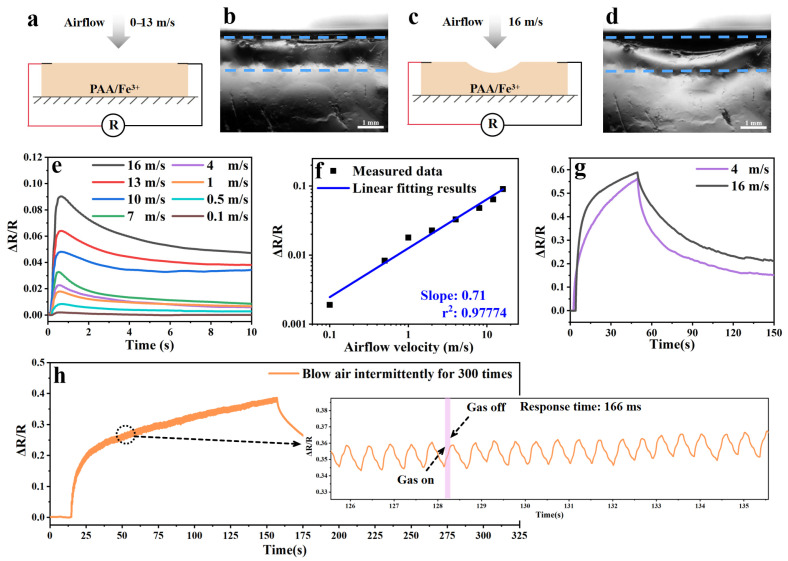
The airflow sensitivity of the PAA/Fe^3+^ hydrogel. (**a**,**c**) A schematic diagram of the action of airflow on the surface of the PAA/Fe^3+^ hydrogel (airflow velocity: (**a**) 0–13 m/s, (**c**) 16 m/s). (**b**,**d**) The side view of the PAA/Fe^3+^ hydrogel surface under the action of air flow captured by the contact angle measuring instrument (airflow velocity: (**b**) 0–13 m/s, (**d**) 16 m/s). (**e**) Resistance responses of the PAA/Fe^3+^ hydrogel under different airflow velocities. (**f**) The linear relationship between the relative resistance variation and the airflow velocity. (**g**) Resistance responses caused by a persistent breeze and strong winds. (**h**) Resistance responses generated under intermittent breezes.

To evaluate the surface sensitivity of PAA/Fe^3+^ hydrogels, we fixed the samples on a test bench and assembled a surface pressure sensor for performance testing; the simulation diagrams are shown in Figure 3a,c. When an air flow (with a velocity of 0–13 m/s) blew across the sample surface, there was no obvious deformation or displacement on the surface (Figure 3b), but a significant resistance change signal response could be detected (Figure 3e). At air flow velocities of 0.1, 0.5, 1, 4, 7, 10, and 13 m/s, the corresponding ΔR/R_0_% values were 0.19, 0.83, 1.78, 2.27, 3.28, 4.80, and 6.39, respectively. As shown in Figure 3f, ΔR/R_0_ is proportional to the air flow velocity. The slope of the linear fit is 0.71 and r^2^ is 0.97774, indicating that the linear model has a high degree of fitting to the data and a strong linear correlation between the two.

When the wind speed increased to 16 m/s, obvious concave displacement changes on the hydrogel surface could be clearly observed in Figure 3d and Video S1, with the ΔR/R_0_% being 9.02. After the continuous blowing of light wind for about 1 min, ΔR/R_0_ reached 0.55, while after the continuous blowing of strong wind for about 1 min, this value rose to 0.6 (Figure 3g). This interesting phenomenon indicates that the sample surface without deformation displacement and the surface with obvious deformation displacement can produce similar signal feedback. The detection range (0.1–16) of the air flow sensor in this study is more advantageous: it not only covers the range of previous studies [32] (0.1–5.5) but also significantly expands the high range, enabling the accurate capture of higher-flow-velocity signals, with stronger adaptability in practical applications, breaking through the limitations of previous studies in high-flow-velocity detection and providing more reliable technical support for monitoring in complex scenarios. To further evaluate the sensitivity of PAA/Fe^3+^ hydrogels, we intermittently blew light wind on their surfaces, and the resulting resistance response is shown in Figure 3h, with an intermittent response time of approximately 166 ms.

Additionally, the PAA/Fe^3+^ sample was tested by physically pressing to induce bulk deformation. As clearly shown in Figure S3, physical extrusion deformation cannot generate obvious response signals in PAA/Fe^3+^ (ΔR/R_0_ = 0.0008), while the signal intensity (ΔR/R_0_ = 0.0328) generated by weak gas flow on the surface is 40 times that of physical extrusion. Based on the presented data, we propose that more significant ion migration occurs on the PAA/Fe^3+^ surface, where fine-tuning the surface state can yield a relatively sensitive response—substantial deformation displacement is not required to generate a strong signal at the surface. This study proposes a surface-responsive ion transport model for re-understanding ion charge transport and fundamental behaviors in hydrogels, hoping to provide new insights into the mechanism of ion migration on hydrogel surfaces.

### 2.3. Ion Transport Mechanisms of PAA/Fe^3+^ Hydrogel

Since the structural changes of materials are used to detect environmental variations, material transport processes must accompany such structural changes. The minimum or optimal transport unit is inevitably molecular-scale material transport. Thus, in this study, removing a small amount of water molecules from the surface of the PAA/Fe^3+^ hydrogel via experimental treatment leads to sensitive changes in the structure that contributes significantly to conductivity at the surface, generating a pronounced concentration effect. As the water content at the surface is relatively low, further reducing surface water content through treatment alters its compactness and order, thereby producing a highly sensitive ion transport response at the surface. During gel formation, the gas–liquid interface inherently provides certain directivity, enabling the formation of interface-directed stable assembly structures. The highest order and regularity of such structures facilitate faster ion migration pathways.

**Figure 4:**
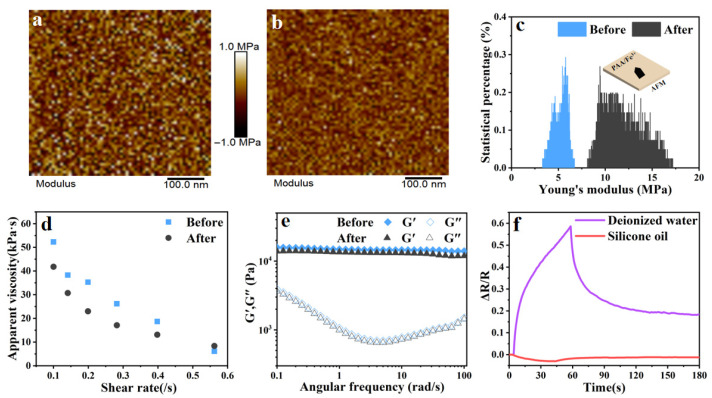
Ion transport mechanisms of the PAA/Fe^3+^ hydrogel. The AFM images of PAA/Fe^3+^ hydrogels before (**a**) and after (**b**) airflow blowing. (**c**) The Young’s modulus of the PAA/Fe^3+^ hydrogel surface before and after airflow blowing was measured by AFM. (**d**) The apparent viscosity of the PAA/Fe^3+^ hydrogel before and after airflow blowing. (**e**) The storage modulus (G′) and loss modulus (G′′) of the PAA/Fe^3+^ hydrogel before and after airflow blowing. (**f**) Resistance responses of the PAA/Fe^3+^ hydrogel at different interfaces after being blown by air following immersion in oil and water.

The surface morphology (Figure 4a,b) and modulus changes (Figure 4c) of the PAA/Fe^3+^ hydrogel before and after air blowing were measured by AFM, revealing significant changes in surface modulus and structure. The Young’s modulus increased from 5 MPa to 10 MPa before and after blowing, which was also verified by the apparent viscosity map (Figure 4d). The rheological properties of PAA/Fe^3+^ before and after air blowing were tested by a rheometer (Figure 4e), showing no obvious differences, indicating that the overall structure of PAA/Fe^3+^ remained unchanged. Its excellent water retention property maintains the stability of the network structure [45,46], and the diffusion of internal water molecules enables the hydrogel’s overall structure to quickly restore stability. The region where PAA/Fe^3+^ changes and exhibits a high-sensitivity response is mainly concentrated on the exposed surface of PAA/Fe^3+^.

To further verify the significant impact of surface water molecule changes on ion migration in PAA/Fe^3+^ hydrogels, the sample surfaces were treated by soaking in water and oil, followed by air blowing tests, as shown in Figure 4f. The water-soaked surface exhibited a better response performance with ΔR/R_0_ = 0.6, higher than that of the unsoaked sample. This is because an abundance of water molecules that accumulated on the surface loosen the structure, reducing the hindrance to ion transport and increasing transmission speed. Under gas flow, the active movement of water molecules generates a more pronounced signal enhancement. In contrast, the oil-soaked surface showed severely degraded signal changes, as silicone oil blocked water molecule diffusion.

In the field of scientific research today, the ion charge transport processes and fundamental behaviors in hydrogel states have always been one of the key topics focused on by many researchers. The hydrogel environment possesses unique complexity, and traditional theoretical models often have certain limitations in explaining ion-related phenomena, making it difficult to comprehensively and accurately reveal the internal mechanisms. In view of this, this study innovatively proposes a surface-based ion transport model. Focusing on the surface region of materials, this model fully considers factors such as the inhomogeneity of charge distribution and subtle changes in intermolecular forces at the surface, verifying the transport trajectories, rate regulation, and charge transfer modes of ions in the quasi-solid phase. Hydrogels have extremely broad application prospects in many emerging technological fields, such as flexible electronic devices, wearable equipment, and biomedical sensors. A deep understanding of the internal logic of ion migration will provide a strong theoretical foundation for technological breakthroughs and product optimization in these fields, facilitating the collaborative and rapid development of scientific research and industry.

### 2.4. Application of PAA/Fe^3+^ Hydrogel

Future smart wearable devices such as intelligent gloves and smart clothing may adopt artificial skin technology, enabling robots’ “skin” to possess pressure-sensing capabilities. This allows them to adjust movements based on the perceived pressure during interaction with the external environment, enabling the more precise grasping and handling of objects while avoiding damage or dropping due to excessive or insufficient pressure [7,47]. In virtual reality (VR) and augmented reality (AR) technologies, physical sensors on artificial skin feedback information such as touch pressure and temperature to users, creating an immersive experience and providing more realistic tactile feedback [6,48,49,50,51]. The resistive change mechanism is widely regarded as the most stable, low-cost, and simplest-structure approach due to its responsive characteristics. Determining how to achieve resistive changes in response to mechanical actions (micro-deformations or gentle breezes) without obvious deformation, similar to human skin, has become particularly important and meaningful.

**Figure 5:**
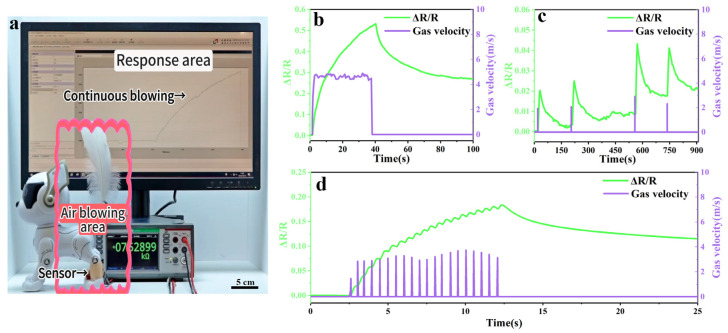
The airflow sensor. (**a**) The robotic dog airflow sensing system. (**b**) Resistance responses of the airflow sensor under sustained airflow velocities. (**c**) Resistance responses of the airflow sensor under single-stream airflow velocities. (**d**) Resistance responses of the airflow sensor under intermittent airflow velocities.

Based on the surface ion transport model and the principle of resistive change, this work may provide promising technical support for conductive polymer hydrogels and intelligent robotic systems with enhanced human–machine interaction sensing functions. To demonstrate the potential of the PAA/Fe^3+^ hydrogel in gas flow sensor applications, we conducted tests on a robotic dog (Figure 5). As shown in Figure 5a and Video S2, the PAA/Fe^3+^ gas flow sensor was placed on the leg of the robotic dog, and a feather was used for the macroscopic observation of gas flow. In the presence of gas flow, the resistance of the PAA/Fe^3+^ sensor increased significantly. Figure 5b–d records the signal responses of the PAA/Fe^3+^ sensor under continuous, single, and intermittent gas flows, respectively. When the gas flow stops, the resistance recovers to a certain extent due to the excellent water retention property of PAA hydrogel maintaining the stability of the network structure. The wearable gas flow sensor based on the surface of PAA/Fe^3+^ hydrogel shows practical application potential for monitoring tiny gas flows, which provides new opportunities for manufacturing simple and flexible artificial skin sensors, holding potential application prospects in the fields of next-generation electronic skin, health detection, and intelligent soft robotics.

## 3. Conclusions

Traditional studies on resistive sensors have mostly focused on bulk ion migration, but this study confirms that when the hydrogel thickness is reduced to the micrometer scale, the surface of the PAA/Fe^3+^ hydrogel regulates network density through humidity under gas flow, generating highly sensitive resistive responses without obvious deformation, breaking through the limitation of traditional sensors relying on mechanical deformation. Through the coordination crosslinking of Fe^3+^ and carboxyl groups of PAA, a three-dimensional network with both mechanical strength and ion transport capability is constructed. While maintaining a tensile strength of 80 kPa and an elongation at break of 1100%, the PAA/Fe^3+^ hydrogel achieves a rapid response (166 ms) to gentle breezes, with its surface response intensity (ΔR/R_0_ = 0.55) far higher than that of bulk deformation (ΔR/R_0_ = 0.0008), verifying the efficiency of the surface ion transport mechanism. A sensing model based on surface ion transport is proposed, revealing the correlation mechanism among surface molecular conformation, water molecule migration, and charge transport in hydrogels, providing a new theoretical framework for the design of resistive sensors. The application of this material in the gas flow monitoring system of robotic dogs has confirmed its potential for practical applications in multiple fields: it shows promising prospects in the fields of intelligent robots and electronic skin, providing technical support for flexible artificial skin sensors; it has potential in the fields of wearable devices and health detection, and can be used in applications such as respiratory monitoring or smart clothing; it can also contribute to virtual reality (VR) and augmented reality (AR) technologies, providing realistic tactile feedback for immersive experiences. This confirms that not only is the sensor theoretically innovative, but it also has practical application value in next-generation electronic skin, intelligent soft robotics, and health detection. In the future, the surface microstructure of the hydrogel can be further optimized (such as introducing nanocomposites or porous designs) to enhance surface ion migration rates; its stability in complex environments (such as alternating humidity and temperature scenarios) can be explored to promote industrial applications.

## 4. Materials and Methods

### 4.1. Materials

Acrylic acid (AA, 99%), iron chloride hexahydrate (FeCl_3_·6H_2_O), and potassium persulfate (KPS, A.R.) were purchased from Aladdin Co., Ltd. (Shanghai, China). Deionized water was used in this study.

### 4.2. Fabrication of PAA/Fe^3+^ Hydrogel

In the presence of Fe^3+^, which acts as crosslinking agents, PAA/Fe^3+^ hydrogels were prepared by in situ free-radical polymerization of acrylic acid, Fe^3+^, and carboxyl groups, and the reaction mechanism is shown in Figure 1. Specifically, AA (3.25 g) was dissolved in deionized water and stirred at room temperature. FeCl_3_ (0.11 g) was added and stirred until a uniform mixture was obtained. The oxygen was removed with the nitrogen for 1 min. Then, initiator KPS (8 mg) was added to the solution and stirred rapidly. The resulting mixture was then injected into a laboratory-manufactured mold consisting of two glass plates with 2 mm rubber gaskets. Finally, these molds were polymerized for 2 h at 50 °C to obtain composite hydrogels. The total volume of the solution mixture was fixed at 30 mL with deionized water.

### 4.3. Characterization

The chemical structure changes of PAA/Fe^3+^ were qualitatively analyzed using a Fourier transform infrared spectrometer (FT-IR, Bruker Optik GmbH, Ettlingen, Germany) with the potassium bromide pellet method, and the set spectral range was 400–4000 cm^−1^.

The X-ray spectra of PAA/Fe^3+^ were obtained through X-ray diffraction (XRD, Bruker D8 ADVANCE, Karlsruhe, Germany), and the scanning range of the diffraction angle (2θ) was 5–60° under 80 mA and 60 kV operating conditions.

A rotational rheometer (Ares G2, New Castle, DE, USA) equipped with a 25 mm diameter rotor (testing distance between the rotor and the substrate: 2 mm) was used to investigate the rheological behavior and apparent viscosity of the PAA/Fe^3+^ hydrogel. The storage (G′) modulus and loss (G″) modulus of PAA/Fe^3+^ hydrogels were obtained as functions of angular frequency ω between the range of 0.1 and 100 rad/s.

The AFM images and Young’s modulus data were acquired using a Bruker Icon atomic force microscope (Bruker Corp, Karlsruhe, Germany).

The side views and videos of the hydrogel being blown by the air flow were captured using a contact angle measuring instrument (Dataphysics OCA20, Filderstadt, Germany). The sample was placed on the stage, and the air flow was blown in the direction perpendicular to the sample, with the macroscopic morphological changes of the sample recorded in real time. The air flow consisted of air.

A tensile test was performed using a HESON machine HS-3004B-S with a 100 N load cell. A hydrogel sample with a 12 mm length × 3 mm width × 2 mm thickness was tested, and the tensile speed was 100 mm/min.

The resistance change data was measured by a multimeter (Keithley DMM6500, Tektronix Co., Ltd., Beaverton, OR, USA).

The relative change in resistance is defined as Equation (1).

ΔR/R_0_ = (R − R_0_)/R_0_ (1)

where R_0_ and R are the original resistance under 0% strain and the real-time resistance under specific strain.

## Data Availability

The original contributions presented in this study are included in the article. Further inquiries can be directed at the corresponding author.

## Supplementary Materials

The following supporting information can be downloaded at https://www.mdpi.com/article/10.3390/gels11080570/s1. Figure S1: The PAA/Fe^3+^ hydrogels with different shapes. Figure S2: The loss factor of the PAA/Fe^3+^ hydrogel. Figure S3: The resistance responses of the PAA/Fe^3+^ hydrogel when exposed to airflow shocks and mechanical percussion. Video S1: Hydrogel deforms when blown by air. Video S2: The airflow sensing system.

## Abbreviations

The following abbreviations are used in this manuscript:

AA	Acrylic acid
PAA	Polyacrylic acid
PAA/Fe^3+^	Polyacrylic acid/ferric ion hydrogel
