# Supplementary material for: Micro-Gas Flow Sensor Utilizing Surface Network Density Regulation for Humidity-Modulated Ion Transport
Source: Gels. 2025 Jul 23;11(8):570. doi: 10.3390/gels11080570 (PMC12385786; doi:10.3390/gels11080570)
Supplement: Supplementary file 1 [file gels-11-00570-s001.zip › SI.docx]

## Supporting Information

# Micro Gas Flow Sensor Utilizing Surface Network Density Regulation for Humidity-Modulated Ion Transport

## Chuanjie Liu^1^, and Zhihong Liu^2*^

^1^ School of Chemistry and Chemical Engineering, Beijing Institute of Technology, Beijing 100081, China

^2^ Shenzhen Polytechnic University, Guangdong 518055, China

^*^ Corresponding author (email: liuzhihong@szpu.edu.cn)


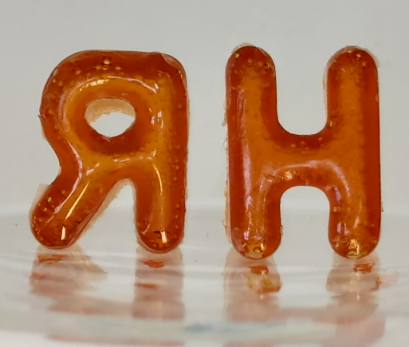


Figure S1. The PAA/Fe^3+^ hydrogels with different shapes.


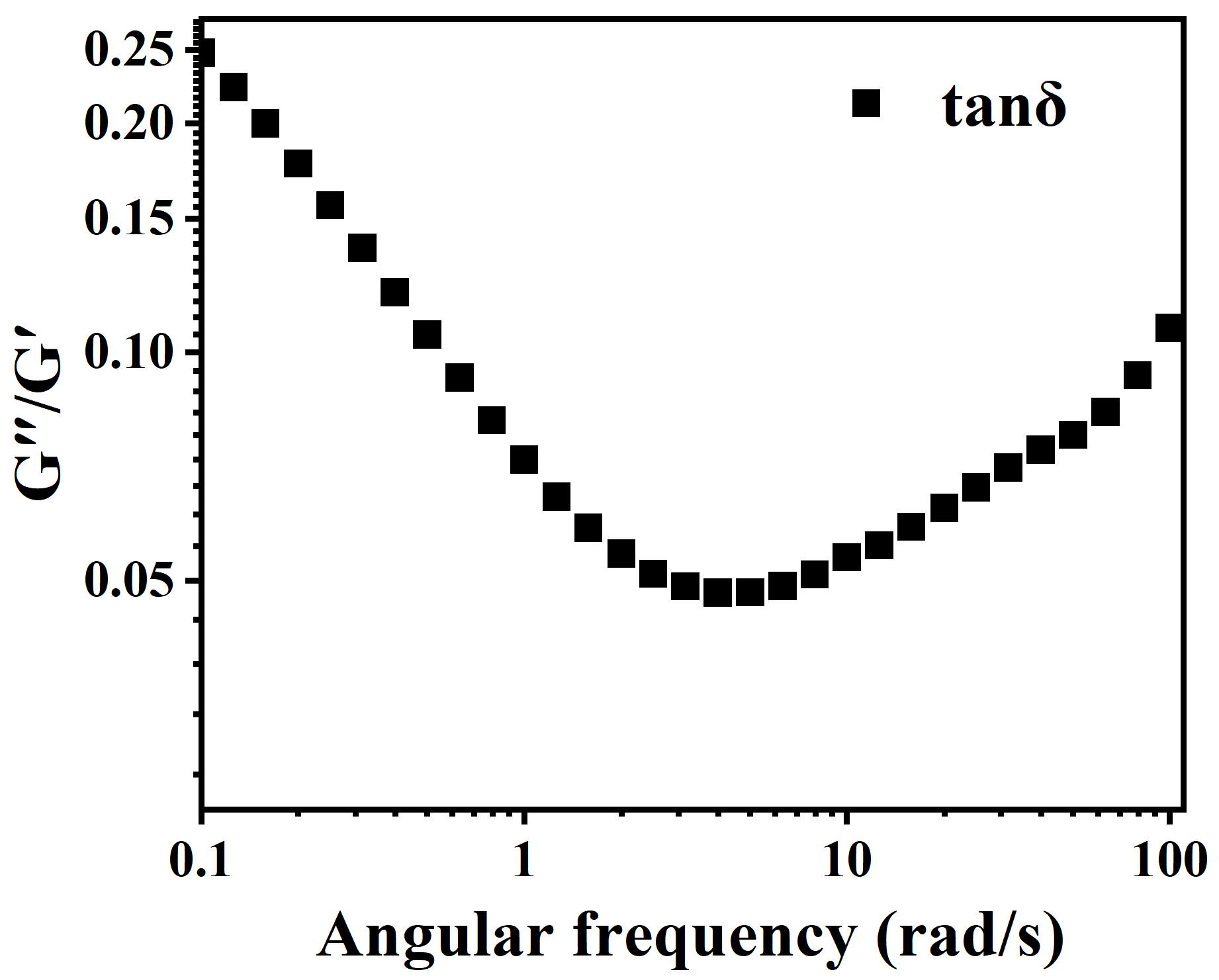


Figure S2. The loss factor of PAA/Fe^3+^ hydrogel.


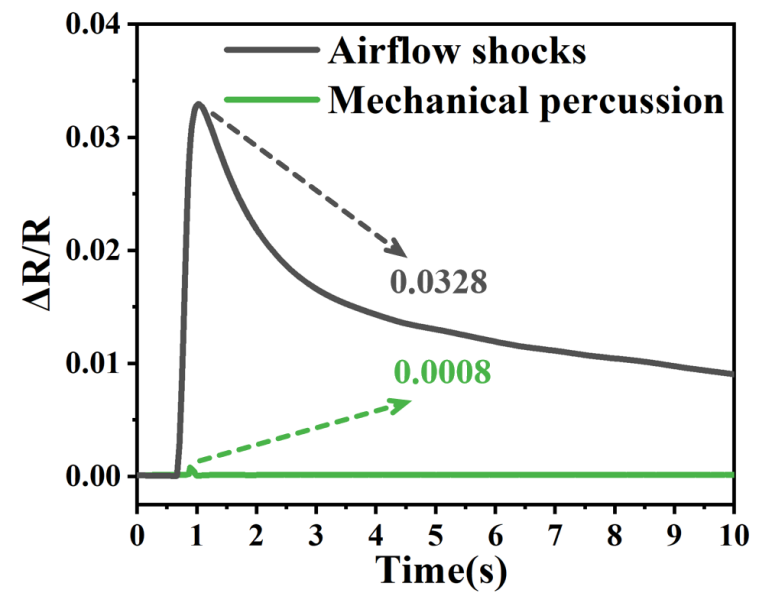


Figure S3. Resistance responses of PAA/Fe^3+^ hydrogel when exposed to airflow shocks and mechanical percussion.
